# Nanoparticle effect on neutrophil produced myeloperoxidase

**DOI:** 10.1371/journal.pone.0191445

**Published:** 2018-01-18

**Authors:** Elodie Sanfins, Alexandra Correia, Stefan B. Gunnarsson, Manuel Vilanova, Tommy Cedervall

**Affiliations:** 1 Biochemistry and Structural Biology, Lund University, Lund, Sweden; 2 NanoLund, Lund University, Lund, Sweden; 3 CEA, Institut François Jacob, Université Paris-Saclay, Fontenay-aux-Roses, France; 4 ICBAS—Instituto de Ciências Biomédicas de Abel Salazar, Universidade do Porto, Porto, Portugal; 5 I3S—Instituto de Investigação e Inovação em Saúde, Universidade do Porto, and IBMC—Instituto de Biologia Molecular e Celular, Universidade do Porto, Porto, Portugal; West Virginia University School of Medicine, UNITED STATES

## Abstract

Nanoparticles affect the immune system as they may interact directly with immune cells and activate them. However, it is possible that nanoparticles also interact with released cytokines and immunologically active enzymes. To test this hypothesis, the activity of myeloperoxidase released from activated neutrophils was measured in the presence of nanoparticles with different chemistry and size. In high concentrations of nanoparticles, myeloperoxidase activity is decreased whereas in low concentrations of nanoparticles the activity is increased. The effect of the nanoparticles on myeloperoxidase is dependent on the total protein concentration as low concentrations of bovine serum albumin together with nanoparticles further increase the myeloperoxidase activity. The results herein show that nanoparticles affect the immune response not only at the cellular level but also on released immune effectors. In particular, they show that the nanoparticle effect on myeloperoxidase activity in the neutrophil degranulation environment is the result of an intricate interplay between the enzyme and protein concentrations in the environment and the available surface area on the nanoparticle.

## Introduction

The exposure to nanoparticles (NPs) in nature and in humans is expected to increase. NPs are intentionally and unintentionally released into nature [1–3], potentially ending up in food webs[4], waterways, and in the atmosphere, for example from combustion of diesel fuel. This leads to exposure routes through food and inhalation. Their actual and potential use in medical applications creates direct exposure to interstitial fluids, blood and airways in humans.

NPs may affect the immune response and immune cells in diverse ways [5,6] and have been reported as immunosuppressor or immunostimulator. As an illustrative example, Fe_2_O_3_ NPs (around 5 nm in diameter) can induce the production of pro-inflammatory cytokines in mice which can trigger the recruitment of neutrophils [7]. Moreover, it has been shown in ovalbumin-sensitized mice treated with a single exposure of iron oxide Fe_2_O_3_ NPs, (around 120 nm in diameter) a marked attenuation of ovalbumin-specific T- and B-cell response [8]. Both of these studies looked at particles of the same composition, but different size and thus different curvature, indicating that besides composition size of the NPs is an important characteristic to consider.

Leukocytes are recruited into inflammatory foci following gradients of chemoattractant molecules that can be produced upon activation of the complement system, by bacterial pathogens or by stromal or tissue resident hematopoietic cells [9]. Release of active enzymes by activated immune cells is an important feature in mediating numerous immune processes that include host defense against invading pathogens [10,11], autoimmune pathology [12], or other inflammatory conditions [13,14]. Neutrophils are particularly important inflammatory cells as they are promptly recruited from the vasculature when inflammation is started but can also mediate the pathogenesis of chronic diseases such as cancer or autoimmunity [15]. In the infected or other inflammatory microenvironments, activated neutrophils may produce reactive oxygen intermediates or degranulate releasing lytic enzymes such as proteases (e.g. elastase, gelatinase, etc.), or myeloperoxidase (MPO) [16]. MPO is an immunologically active enzyme that catalyzes the oxidation and chlorination of macromolecules using hydrogen peroxide as a substrate to form hypochlorous acid adducts [17–20]. Granule enzymes may be released to the extracellular space or become associated with neutrophil extracellular traps (NETs) where they can interact with any macromolecules present such as pathogen membrane proteins [21]. NPs present in the organism can then interact with the neutrophil exocytosed proteins.

In a biological fluid, NPs bind proteins forming a protein corona [22,23]. The interaction with the nanoparticle surface will affect protein structure and function [24–28]. The effect on the protein is dependent on the surface chemistry and the curvature, i.e. the size of the nanoparticle [24,25,29]. Enzymatic activity is one protein function that has been demonstrated to be influenced by NPs surfaces [27]. Factor XII in the blood coagulation is inhibited, activated, or unaffected by negatively charged polystyrene NPs depending on their surface chemistry and curvature [30]. The effect on Factor XII activity depends on the overall protein concentration as crowded surfaces affect the binding of proteins. Possibly because the structural change and orientation can be different depending on the available surface area. Consequently, the NPs impact on enzyme can be different in low protein conditions, for example, using only purified proteins compared to high protein concentrations as in blood. The influence on protein structure and function by nanoparticle surfaces suggests that NPs can affect the immune response by activating immune cells or by interacting and affecting the released enzymes from the activated immune cells. Not only protein`s interactions with NPs are immunologically important. Other biomolecules such as lipopolysaccharide and glycan can, after absorption to the NP`s surface, modulate the immune response to themselves [31,32]

Here, we have studied MPO activity in the presence of NPs with different chemistry and size. We used polystyrene NPs with sizes between 25 and 220 nm and with different surface chemistry, sulfon (OSO_3_), carboxyl (COOH) and amine (NH_2_), Table 1. This set of NPs makes it possible to compare the effects of size of particles with the same surface chemistry and the effect of differently charged modifications on the surface on nanoparticles with the same size. TiO_2_ was added as metal oxide particle with a different surface chemistry and dispersion stability. The obtained results show that MPO activity is affected by the particle surface and by the presence of other proteins. Bovine serum albumin (BSA) decreased the MPO activity in solution but together with particles BSA in low concentrations increased the MPO activity indicating that the interaction between MPO and the particles surface is different depending on the available surface area.

**Table 1:** Characteristics of the used nanoparticles.

Particles with manufacturers diameter (nm)	Diameter (nm)^1^			
	H_2_O	PBS	Media^2^	Medium^2^ + 10% FCS
PS-COOH (26)	30.5 ± 0.1	28.3 ± 0.3	28.4 ± 0.1	57.9 ± 0.5
PS-COOH (60)	65.4 ± 0.2	59.3 ± 0.4	58.2 ± 0.5	120 ± 1
PS-COOH (220)	211 ± 1	205 ± 1	205 ± 2	258 ± 3
PS-NH_2_ (5756)	49.5 ± 0.1	245 ± 7	52.7 ± 0.4	243 ± 2
PS-NH_2_ (120)	111 ± 1	161 ± 3	118 ± 1	142 ± 1
PS-OSO_3_ (25)	29.4 ± 0.2	27.7 ± 0.2	28.6 ± 0.2	71.8 ± 1.2
TiO_2_ (5)	403^3^	603^3^	^4^	^4^

^1^Determined by DLS of 0,1 mg/ml nanoparticles

^2^RPMI

^3^Determined by DSC

^4^Not Determined.

## Material and experiments

### Proteins, serum and nanoparticles

MPO was purchased from Abcam, UK, and was dissolved in PBS, 100 μg/ml, and frozen in aliquots. BSA was purchased from Sigma and dissolved in PBS 35 mg/ml and frozen in aliquots. Cow serum was purchased from Sigma or Innovative Science, USA and fetal calf serum (FCS) from Innovative Science, USA. Both sera were initially defrosted, heat inactivated at 56°C for 30 min and frozen in aliquots. All polystyrene particles were purchased from Bangs Inc, USA, and TiO_2_ particles, anatase phase, from PlasmaChem, Germany Cat Nr PL-TiO-5p-10gr.

### Characterization of size of particles and nanoparticles/protein complexes

The dynamic light scattering of 100 μl samples in 96 well non-treated polystyrene Costar® assay plate, Corning, USA, was obtained on a DynaPro platereader-II, Wyatt Technology, USA, at 25°C, with 20 seconds accession time, 10 accessions per sample. All samples were measured in triplicate. The size of used NPs was determined in H_2_O, PBS 1× (phosphate buffered saline), RPMI medium, RPMI supplemented with 10% FCS and the obtained light scattering data were analyzed using the cumulative mode assuming one population of particles. Differential centrifugal sedimentation (DCS) was performed on DC24000 disc centrifuge (CPS Instruments, USA), with a linear 8–24% sucrose gradient at 23,677 RPM.

The sulfon (PS-OSO_3_) or carboxyl (PS-COOH) modified polystyrene NPs have similar size in H_2_O, PBS, and RPMI medium, whereas the amine (PS-NH_2_) modified polystyrene and TiO_2_ NPs increase in PBS, Table 1. The size of all polystyrene NPs increase in 10% FCS indicating the formation of protein corona and/or aggregation. The size of TiO_2_ NPs at different particle and cow serum concentrations are further analyzed below. The zeta potential is negative for the PS-COOH, PS-OSO3, and TiO_2_ in PBS, and positive or neutral for the PS-NH_2_, Table A in S1 Appendix.

### Cell culture and activation

Human peripheral blood was obtained from healthy donors. Experiments involving human blood and cells have been approved by the Ethics committee of ICBAS (document 172/2016). The study was conducted according to the principles expressed in the Declaration of Helsinki. Written informed consent was obtained from all study participants. Neutrophils were separated by centrifugation using Histopaque-1119/1077 gradient, Sigma Aldrich. Cells were washed with PBS containing 2 mM EDTA with at least 3 volumes of buffer. Remaining erythrocytes where then lysed with an ACK solution (Ammonium-Chloride-Potassium) and neutrophils washed again with PBS containing 2 mM EDTA. The neutrophil pellet was resuspended in RPMI-1640 medium supplemented with Penicillin (200 IU/ml) and Streptomycin (200 g/ml), L-Glutamine (4 mM), β-mercaptoethanol (0,05 mM) (all from Sigma), and with 10% FCS, Biowest, France or without serum when mentioned. Cells were then seeded in 96-well round-bottom culture plates (Nunc, Denmark) at 10^6^ cells per well. 40 ng/ml of phorbol myristate acetate (PMA, from Sigma Aldrich) was added to each well to induce the release of MPO during 4h. The media and cells were pooled and centrifuged at 300 g for 10 min. The supernatants of activated neutrophils were immediately used for activity assays or kept frozen at -80°C for further experiments.

### MPO activity assay in cell supernatant

100 μl of supernatant of activated neutrophils or 1 μg MPO in 100 μl PBS, were mixed with NPs in a concentration of 0,25 to 1 mg/ml and incubated at 37°C for 30 min. 50 μl of this reaction were then added to two different wells in a 96-well plate. Freshly prepared 0,03% H_2_O_2_ solution in 100 mM NaAcetate, pH 5.2 (100 μl per well) was added followed by the addition of 100 μl of tetramethylbenzidine (TMB) ready to use substrate (from Thermo fisher Scientific). The MPO activity was measured by following the cleavage of TMB substrate changing color to blue (followed at 605nm wavelength) when H_2_O_2_ is reduced by MPO. The reaction was then monitored on a plate reader on kinetic mode for 30 min with an O.D. measurement at 605 nm wavelength every 30 s.

In the competition experiments 1 μg MPO were mixed with BSA in 100 μl PBS. The final BSA concentration was between 1 and 36 mg/ml. PS-COOH, 60 nm, NPs were added to a concentration of 0.1 mg/ml. PBS was added to control samples without NPs. The MPO activity was measured as above.

### Separation and characterization of bound and unbound MPO

The direct interaction between MPO and NPs was analyzed by mixing MPO and nanoparticles in PBS, and after 1 hour incubation, separating unbound MPO from bound by centrifugation, 13000 g, for 30 to 60 min. Thereafter, the supernatant was analyzed for MPO activity (see above). The MPO concentration was 1 μg/ml and the nanoparticle concentration 0,1 mg/ ml. In another set of experiment, 1 mg NPs were mixed with 1 or 2 μg of MPO in a total volume of 21 μl PBS. After centrifugation, the presence of MPO in the supernatant was determined by 10% SDS-PAGE electrophoresis. High Molecular Weight standard, NZYColour Protein Marker II (nzytech, Lisbon, Portugal) was used to determine protein mass upon silver staining. In both experiments, the formed pellet was not stable which made it difficult to wash and analyze it. Band quantification was implemented with ImageJ software with the embedded gel analysis tool.

The interaction between MPO and NPs were further analyzed by loading the mixtures of MPO and NPs or only MPO or only NPs on top of a stepwise sucrose gradient. The first layer was 10% sucrose in PBS and the second layer 40% sucrose in 50:50 PBS and TMB. The MPO NPs mixtures were centrifuged at 18000 rpm for 30 minutes. The NPs with or without MPO are captured at the 40% sucrose layer containing TMB. The color reaction was photographed after 1 h incubation.

### Statistical analysis

Results are expressed as mean values ± standard deviation. Data were analyzed by one-way ANOVA followed by Holm-Sidak's multiple comparisons test. Data were considered significant at p<0.05. GraphPad Prism version 6.01 (GraphPad Software, SanDiego, CA, USA) for Microsoft^®^ Windows was used for statistical analyses

## Results and discussion

One of the challenges in nanotoxicology studies is the understanding of the direct impact of nanoparticles in a complex environment. Studies focused on the effect of particles on protein function could lead to the deciphering of the net impact of nanoparticles on biological pathways.

MPO mediates resistance to infectious agents as it is involved in both intracellular and extracellular [21,33] killing of a wide range of pathogenic agents such as bacteria, fungi or viruses. As observed, naturally occurring, MPO mutations in human can induce [34] structural modifications in this enzyme that could affect its activity. Thus, MPO may have implications in host physiology and the immune response, highlighting the importance of this protein in some disease conditions. Patients with low or non-active enzymes in phagocytic vacuoles will be deficient in microbial killing and will develop recurrent and severe infections. For example, low or totally absent activity in NADPH oxidase of phagocytes is responsible for a clinical condition designated as chronic granulomatous disease [35]. NADPH oxidase produces superoxide free radical converted into hydrogen peroxide; MPO catalyze the production of hypochlorous acid using hydrogen peroxide and chloride anion [36]. The effect of NPs on MPO would mimic a chronic enzymopathy during these crucial steps of microbial killing.

**Fig 1:**
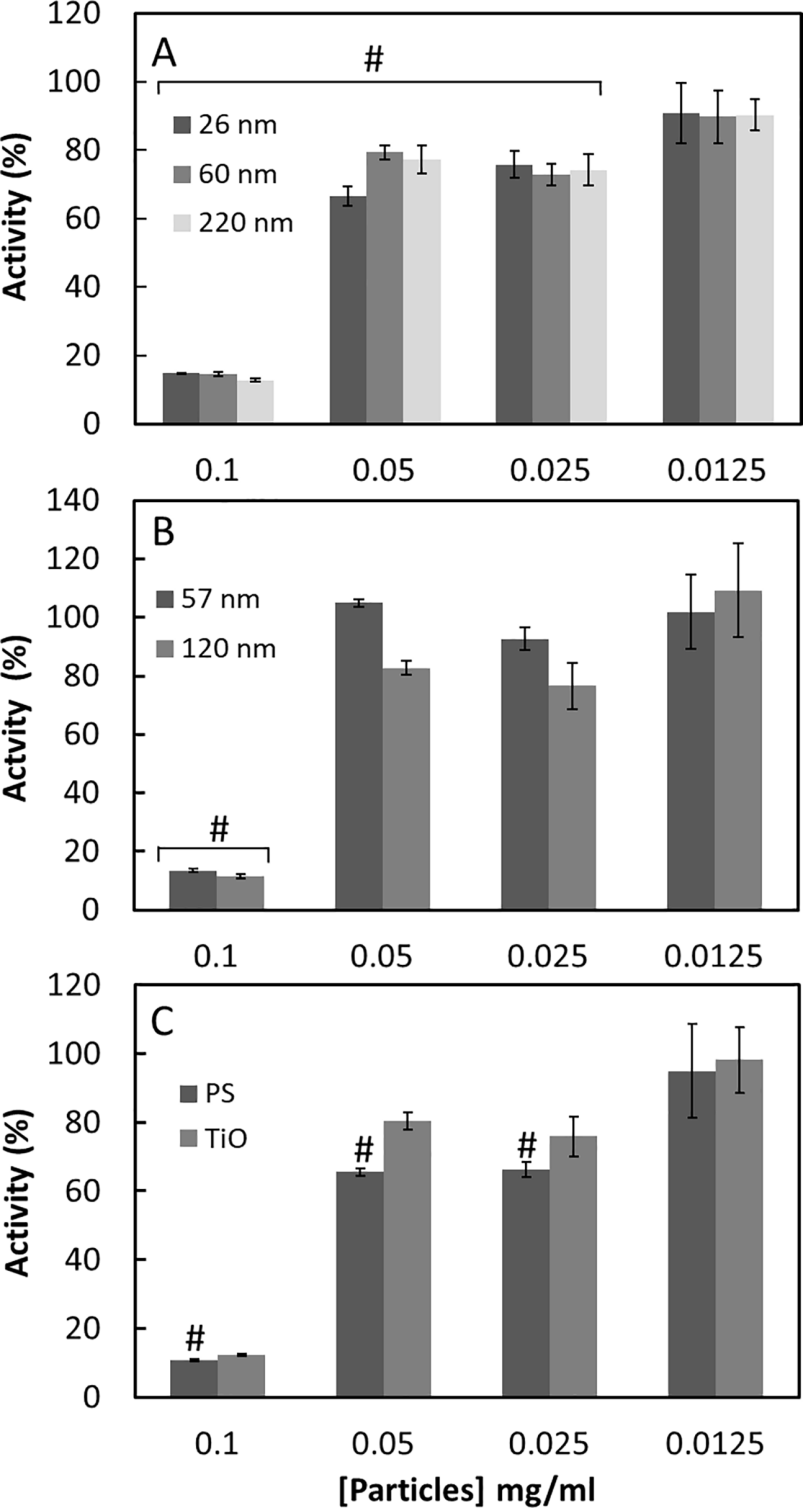
The effect of nanoparticles on secreted MPO in serum free media. A- PS-COOH NPs; B- PS-NH2 NPs; C- PS-OSO3 or TiO_2_ NPs, as indicated. The supernatants from activated neutrophils were analysed for MPO activity. The results are reported as % of MPO activity in supernatants without added nanoparticles. The results are a mean of three separate experiments each done in duplicate. (#)p < 0,05 compared to control without NPs.

First, neutrophils prepared from freshly collected blood were incubated in serum free medium and activated by PMA to stimulate granule release and thereby the release of MPO. The MPO activity was measured by following the cleavage of the chromogenic substrate changing color (to blue) when hydrogen peroxide is reduced by MPO. The change of colour was followed at 605 nm. The effect of NPs on the MPO activity was evaluated by adding different amount of polystyrene NPs with different size, surface chemistry and charge, and TiO_2_ NPs to culture supernatants of PMA-stimulated neutrophils. (See NPs characteristics in Table 1) As the MPO concentration in the culture supernatants is not known, we report the effect of NPs on the MPO activity as the percentage of control (no added nanoparticles—100% MPO activity). Fig 1A shows that negatively charged PS-COOH nanoparticles inhibit the MPO activity. Although the particles are of different size and thereby exhibit very different surface area at the same mass concentration, strong inhibition (more than 80%) is only seen at 0.1 mg/ml. Fig 1B shows the effect of PS-NH_2_ NPs on the secreted MPO activity. As for the PS-COOH NPs, there is an inhibition at 0.1 mg/ml of NPs. Furthermore, there is no size dependency of the inhibition. PS-OSO_3_ NPs and TiO_2_ particles, Fig 1C, also inhibit the MPO activity at the highest concentration.

**Fig 2:**
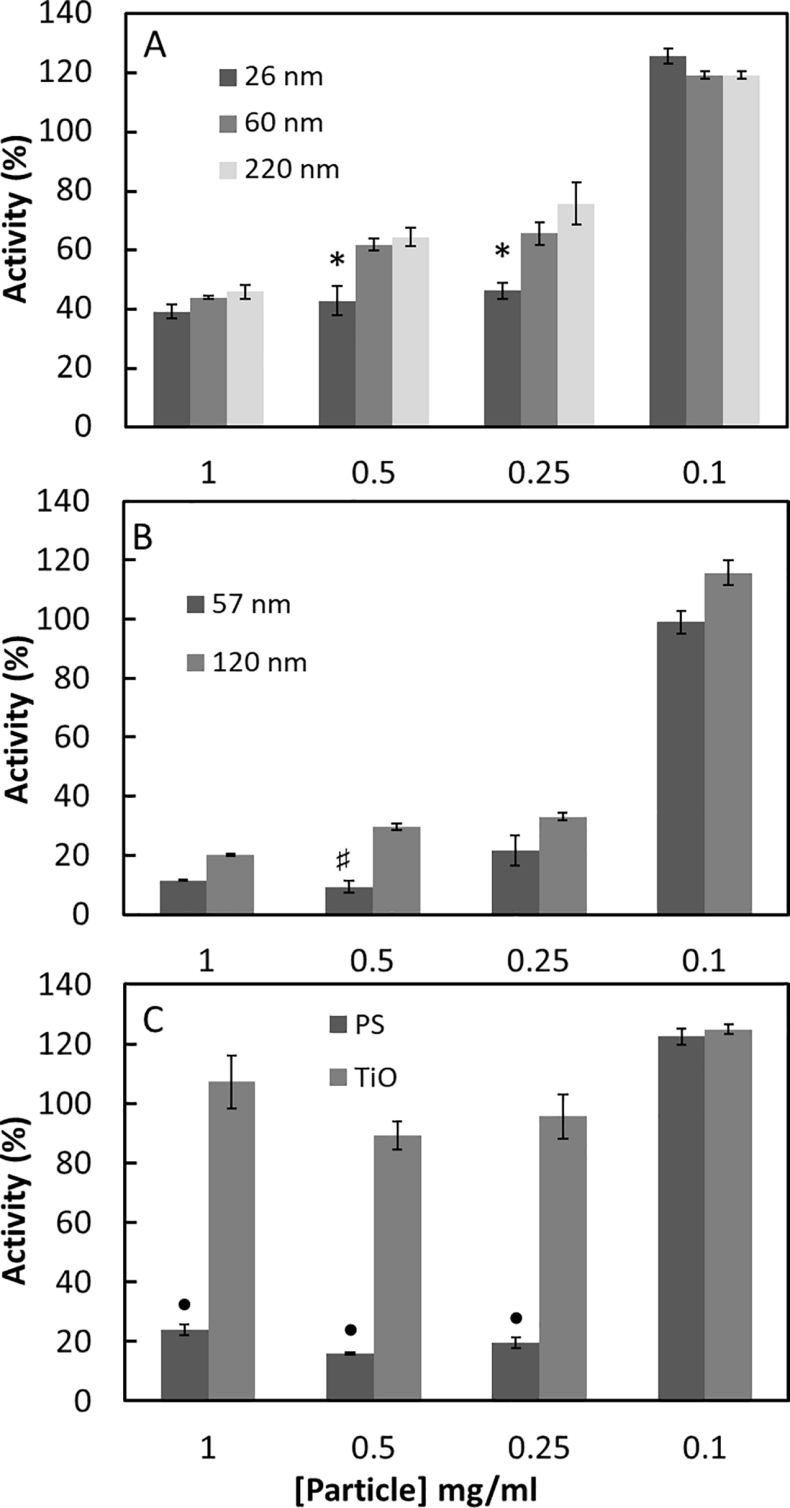
The effect of nanoparticle on secreted MPO in protein rich media. A- PS-COOH NPs; B- PS-NH2 NPs; C- PS-OSO3 or TiO_2_ NPs, as indicated. The supernatants from activated neutrophils were analyzed for MPO activity. The results are reported as % of MPO activity in samples without added nanoparticles in protein rich media. The results are a mean of three separate experiments each done in duplicates. p< 0,05 for all concentration of nanoparticles versus 0,1 mg/ml, expect for enzyme activity in presence of TiO_2_ which is not significantly different for all TiO_2_ concentration* p<0.05 versus activity in presence of 220nm COOH NPs. #p<0.05 versus activity in presence of 120 nm NPs. ● p<0.05 versus activity in presence of 26 nm, 60 nm, 220nm COOH NPs, 120 nm NH_2_ NPs and TiO_2_ NPs at 1, 0.5, 0.25 mg/ml.

**Fig 3:**
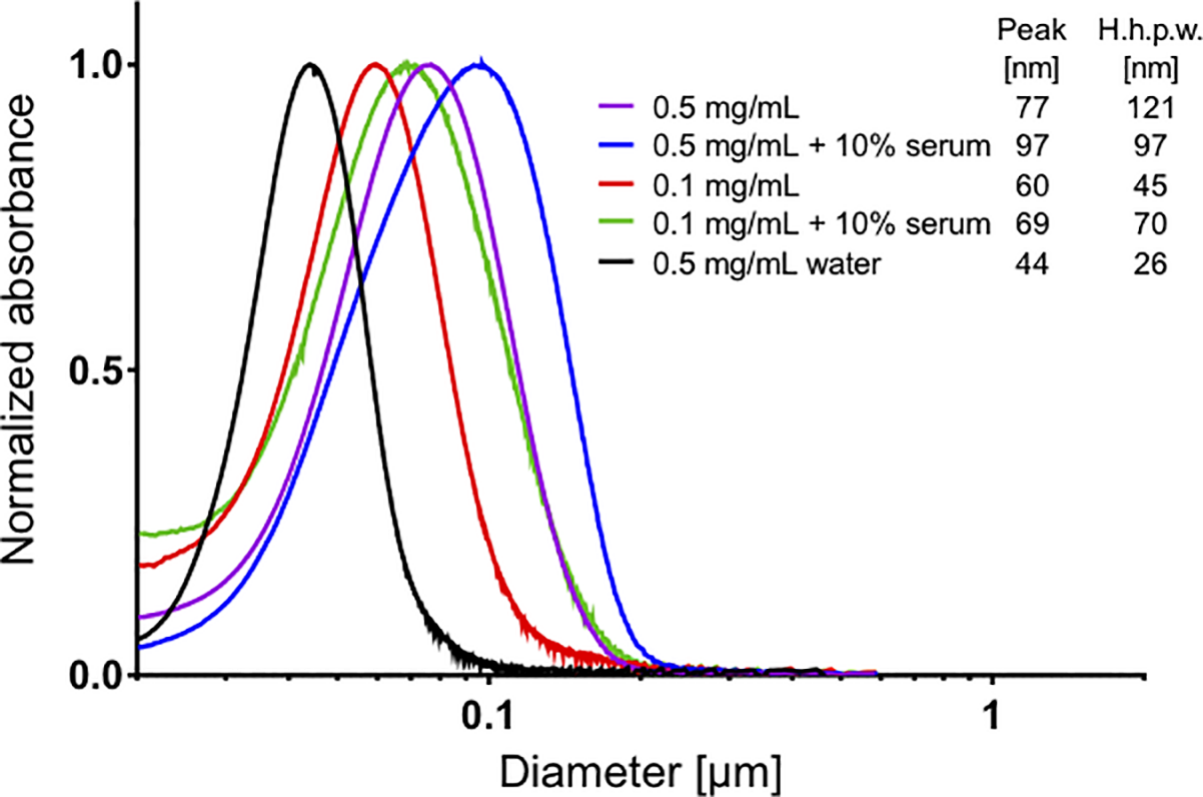
The size of TiO_2_ particles in PBS or PBS with 10% serum. The peak diameter is defined as the diameter value where normalized absorbance is 1, and the half height peak width (H.h.p.w.) is defined as the difference between the two points where normalized absorbance is equal to 0.5.

Many cell assays and toxicological tests are performed in medium containing fetal calf serum (FCS), which makes the medium protein rich. As in the serum free experiment, the MPO activity is inhibited by the polystyrene particles although an increase in concentration is needed going from 0.1mg/ml to 1mg/ml, Fig 2. However, smaller particles inhibit the MPO activity more than larger particles, most likely due to the larger surface area. In protein rich conditions, a larger surface area is probably needed because other proteins compete with MPO for the particle surface. Interestingly, at the lowest particle concentration tested the MPO activity is increased. Moreover, in the protein rich media the lowest concentration increases the activity of the MPO (120% of control activity, without added NPs) while in protein poor media 0.1mg of any NPs tested in this study was enough to decrease the activity of MPO by 80%. One of the NPs tested, TiO_2_, does not inhibit the activity. This may be due to aggregation of TiO_2_ particles in protein rich environment effectively decreasing available surface area. However, at low particle concentrations the MPO activity is increased just as for the polystyrene particles. The aggregation of TiO_2_ in PBS and PBS with 10% serum will depend on the particle concentration. Fig 3 shows the size measured by differential sedimentation ultracentrifugation, at two different concentrations of TiO_2_ in PBS. Compared to in water the aggregate size is larger and the size distribution broader. Both parameters increase when the TiO_2_ concentration increases. Adding 10% FCS further increase the aggregate size. These data show that the aggregation of TiO_2_ is different in different concentrations and different ratios of protein and particles, which makes a concentration dependent effect of TiO_2_ in protein rich environments difficult to evaluate.

In Fig 2, there is a clear difference between the effects of particles with different surface chemistry. The 25 nm PS-OSO3 NPs inhibit the MPO activity, in presence of serum, more than similar sized PS-COOH NPs. These particles both have Z-potential charge but their surface chemistry differs. Differences in the extent PS-COOH and PS-OSO3 NPs affecting enzymatic activity have been reported before, such as on the proteolytic activity of coagulation factors. Small PS-COOH NPs inhibit the coagulation whereas it is unaffected by PS-OSO_3_ NPs [30,37]. The inhibitory effect is directly on Factor XII [30]. Another difference due to the surface chemistry is seen when comparing the effect of 60 nm PS-COOH and 57 nm PS-NH2 NPs on MPO in serum as the amine modified PS-NH2 inhibitory effect on MPO activity is stronger, Fig 2.

**Fig 4:**
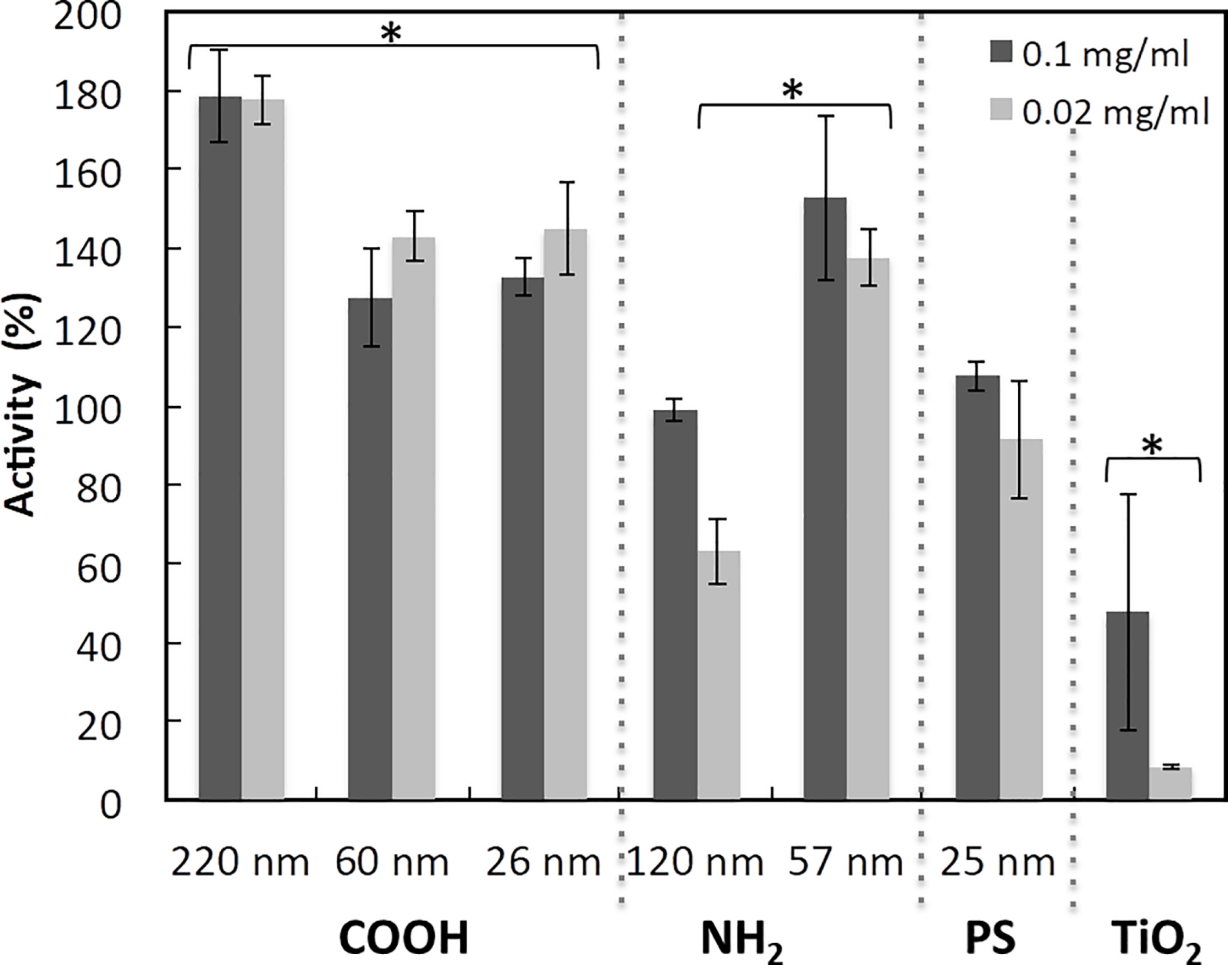
The effect of nanoparticles on purified MPO. Purified MPO in PBS was mixed with the different particles before the MPO activity was measured. The results are reported as % of MPO activity in samples without added nanoparticles. * p < 0,05 versus enzyme activity without NPs.

The above results suggest, that depending on the conditions MPO activity can be either increased or decreased by the particle surface. As the conditions in the cell media are complex, we wanted to study the effect on MPO in a controlled environment using purified protein. Fig 4 shows how the different particles at two different concentrations affect purified MPO activity. All PS-COOH nanoparticles increase the MPO activity in PBS without FCS proteins or any other protein neutrophils secrete, that could interact with the NPs surface. The increase in activity is most pronounced for the 220 nm PS-COOH NPs. In the same mass concentration, the available surface area is lower for the 220 nm than for the 60 and 26 nm PS-COOH NPs. This indicates that it is not only the available surface area that determines the effect on the MPO but also the curvature. As higher concentrations of particles decreased the MPO activity in the cell media, it would have been interesting to see how higher particle concentration affect the activity of the purified enzyme. However, further increase of the particle concentration induced aggregation, which makes the analyses of enzyme activity impossible. The 57 nm PS-NH_2_ nanoparticle increase the MPO activity whereas the 120 nm decrease the activity. This is not only due to available surface area as the surface area in 0.1 mg/ml 120 nm particles is approximately the same as the surface area in 0.02 mg/ml 57 nm particles, indicating that the curvature effect is opposite for the amine modified particles compared to the carboxyl modified particles. The TiO_2_ particles decrease the MPO activity. Interestingly, the decrease is more pronounced in lower particle concentration, probably due to particle aggregation and reduced available surface area.

**Fig 5:**
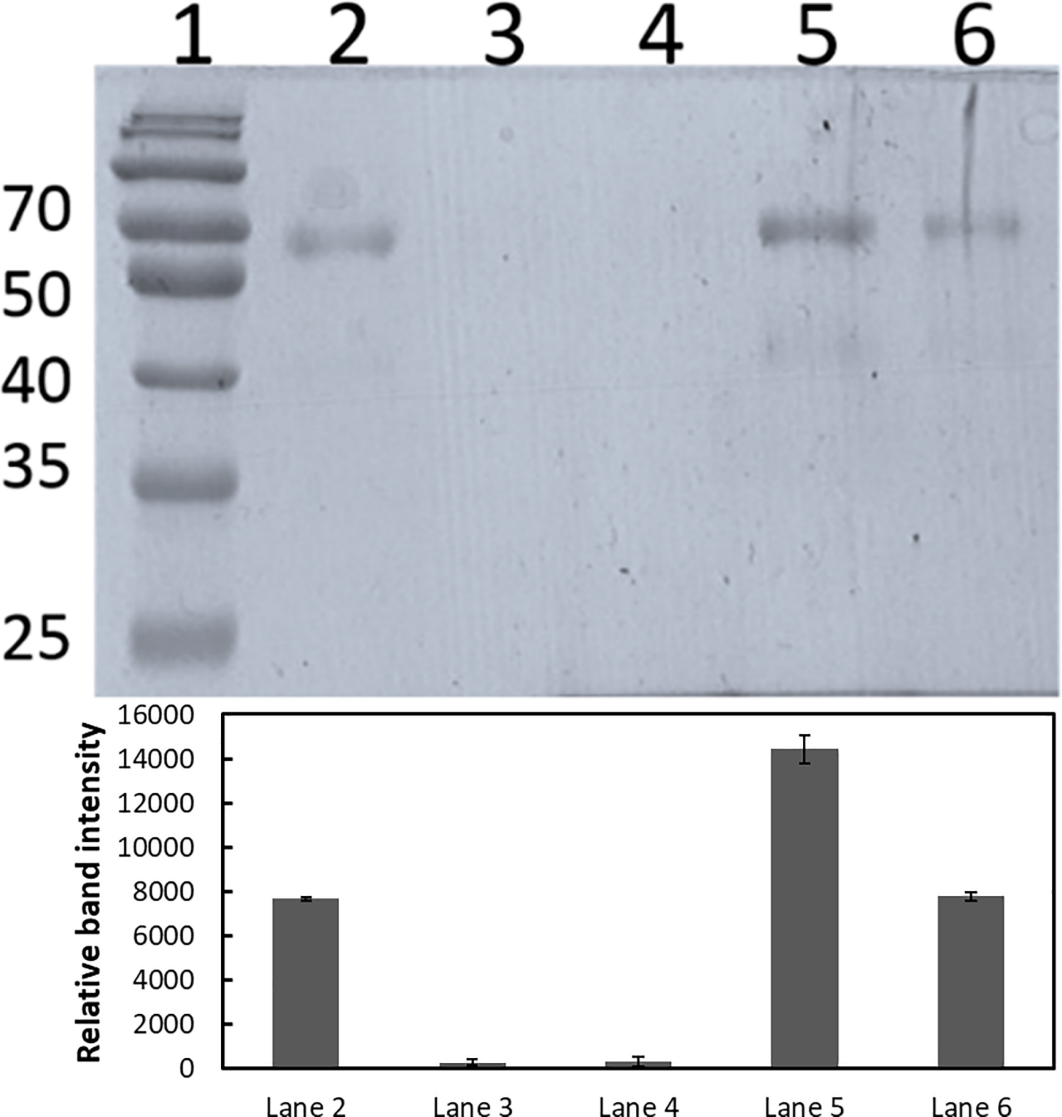
Interactions between 60 nm PS-COOH NPs and MPO evaluated by SDS-PAGE. Lane 1: High Molecular Weight standard (kDa), lane 2: 0.5 μg MPO, lane 3–6: supernatant after centrifugation of 2 μg MPO (lanes 3 and 5) or 1 μg (lanes 4 and 6) mixed with 1 μl (1 mg) 60 nm PS-COOH (lanes 3 and 4) or with 1 μl H_2_0 (lanes 5 and 6). Image J software was used to estimate the bands intensity and is represented as relative intensity for each lane (or relative quantity for each lane taking the first lane equal to 0.5μg/ml of MPO).

The data show that MPO is affected by the NPs surface. A likely explanation is that there is a direct interaction between MPO and particles. All NPs were tested for MPO binding by adding MPO particles mixtures on top of a stepwise sucrose gradient. In the top sucrose is 10% in PBS and the bottom 40% in PBS and TMB, the MPO substrate. After centrifugation the MPO NPs complexes are trapped in the 40% sucrose, which contain TMB. The presence of MPO is detected by the colour reaction. In this assay all NPs interacted with MPO, Figure A in S1 Appendix. Thereafter we used three additional approaches to confirm the interaction between the 60 nm PS-COOH NPs and MPO. First, 2 μg or 1 μg MPO were mixed with 1 mg of 60 nm PS-COOH particles. After incubation the protein/particle complexes were pelleted by centrifugation and the presence of MPO in the supernatants detected by SDS-PAGE electrophoresis (Fig 5). The MPO is depleted from the supernatant indicating that MPO interacts strongly with the 60 nm PS-COOH NPs. Second, we mixed 1 μg/ml MPO with 0.5 or 0.05 mg/ml of 60 nm PS-COOH particles, in the same conditions used to study the effect of PS-COOH on MPO activity (see Figs 4 and 5). The mixtures were centrifuged and the MPO activity in the supernatants assessed. The activity in the supernatants is lower than in the control samples (Table 2) again indicating that MPO is depleted from solution as the particles are pelleted. The activity is higher in the samples with more particles. The explanation for this is that in both centrifugation assays the formed pellets are loose and MPO/particle complexes may still be in the supernatant. As MPO activity is increased in the presence of particles, the effect of a small amount of MPO can give a relatively large signal. Third, the size of 60 nm PS-COOH particles in the presence of MPO was determined. The size of the 60 nm PS-COOH particles is similar with or without MPO, Table 2, which shows that in this concentration range of MPO and particles, no particle aggregation occurs.

**Table 2:** Size of the 60 nm PS-COOH nanoparticles with or without MPO and MPO activity after removal of particles by centrifugation.

[Particle] (mg/ml)	Diameter (nm)		Activity in supernatant
	Without MPO	With MPO	(% of MPO activity)
0.5	58.8 ± 0.2	59.2 ± 0.7	43 ± 3
0.05	/	/	14 ± 12
0.02	60.6	61.4 ± 0.2	/

**Fig 6:**
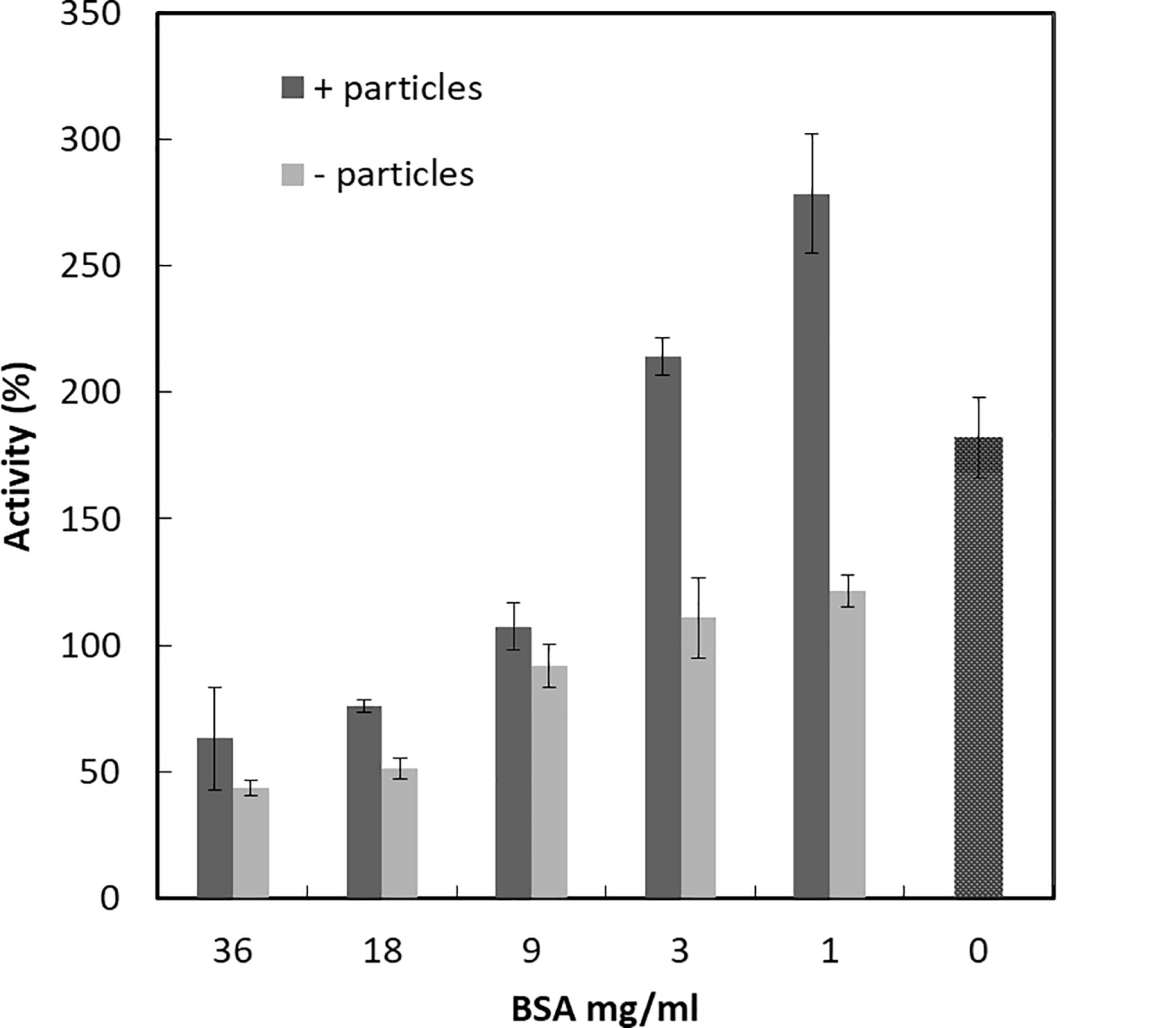
The combined effect of bovine serum albumin alone and albumin with 60 nm PS-COOH on MPO activity. MPO was mixed with different concentrations of BSA and thereafter with 60 nm PS-COOH before the MPO activity is measured. The results are reported as % of MPO activity in samples without added BSA or nanoparticles. All activities in presence of NPs with or without added BSA are significantly different from activity without BSA (p<0.05).

Next, we wanted to investigate how the competition of other proteins affects the impact of NPs on MPO activity. We chose to use 60 nm PS-COOH particles and purified bovine serum albumin (BSA). BSA is the most abundant protein in FCS and cow serum, moreover, approximately half the protein mass is albumin in blood serum. Fig 6 shows how the activity of MPO is affected by increasing BSA concentration with or without 60 nm PS-COOH particles. At high BSA concentration, the MPO activity is decreased. With particles alone, the activity is as before increased. In the presence of 1 and 3 mg/ml BSA, the activity is further increased and reaches a maximum at 1 mg/ml. At high BSA concentrations, the particles effect seems to be weakened. To confirm the presence of MPO on the particles we used the same stepwise sucrose gradient as above. MPO together with different concentrations of BSA was mixed with the 60 nm PS-COOH NPs and loaded on top of the gradient. After centrifugation and incubation, the blue color on top of the 40% sucrose layer indicates the presence of MPO. As can be seen in Figure B in S1 Appendix, MPO is present on the NPs in BSA concentrations from 0 to 9 mg/ml. These results support previous data in protein rich cell media. There is an increased activity of the MPO depending on the surface area and on the amount of the surrounding proteins on the surface.

We have previously seen that small 26 nm PS-COOH NPs inhibit factor XII coagulation activity whereas larger PS-COOH NPs increase the factor XII activity. The size threshold seems to be around 60 nm for PS-COOH and somewhat smaller for silica and metallic particles. The MPO activity is not clearly affected by curvature in our experimental conditions and the observed differences can be explained by differences in the exposed surface area.

However, two differences are seen that can be due to surface curvature. First, pure MPO is further activated by the 220 nm PS-COOH than by the 26 and 60 nm PS-COOH although the smaller particles present more surface area, indicating that a small surface curvature activates MPO more than a large surface curvature. Second, the activity of pure MPO in the presence of 120 nm PS-NH2 NPs is lower when compared with 57 nm PS-NH2 NPs. This effect cannot be explained by differences in surface and may reflect that larger particles with smaller surface curvature activate MPO less or do not activate MPO at all. Interestingly, differences in surface chemistry result in opposite effects of the surface curvature. This clearly shows that it is difficult to predict how particles of different material and size will affect enzyme activity and stress the need of experimental evaluations. The effect on MPO activity by the studied NPs is complex. It seems to be determined not only by the surface curvature and chemistry but also by an intricate interplay between available surface and the presence and competition of other proteins. Several polystyrene particles increase the activity of purified MPO. This can be explained by a direct interaction between the enzyme and the particle surface that change the structure or stabilize the MPO structure. This has been seen for several other proteins in the presence of NPs [24,26,27,29,38].

Another explanation is that putative weak distant interactions between the enzyme and the surface and between the substrate and the surface would increase the effective concentrations and reduce the diffusion distances. A weak interaction between the substrate and the surface will also increase the activity if the enzyme is directly bound to the surface. Evidently, MPO interacts strongly with the PS-COOH NPs, which suggests that the interaction stabilizes the enzyme and/or induces a favorable structure. We have no indications of interactions between the substrate and the surface but it cannot be ruled out.

**Fig 7:**
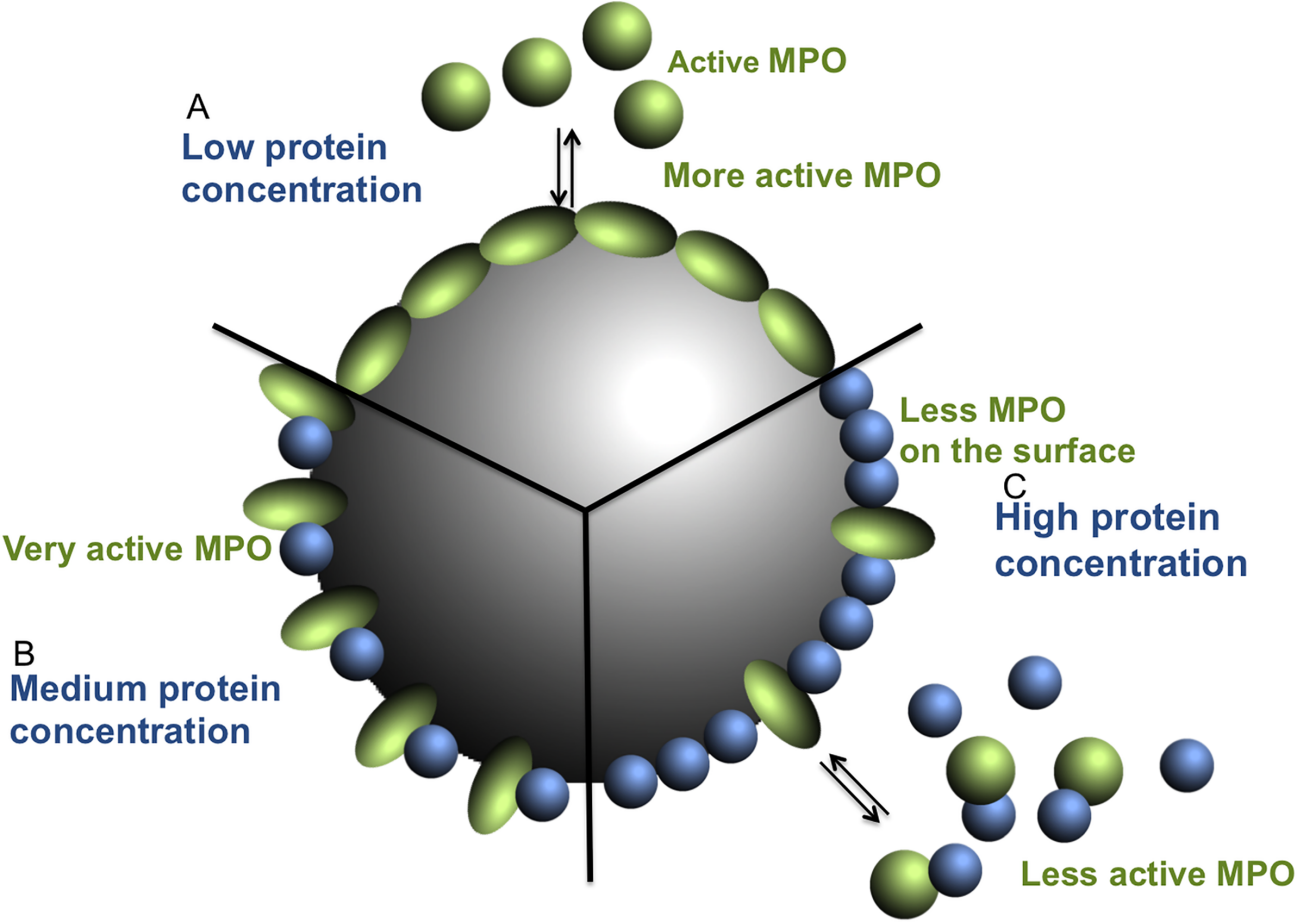
Schematic representation of how MPO (green), particle surface and BSA (blue) concentration interplay to define the activity level of MPO. (A) When purified MPO interacts with the particle surface its interaction modifies the conformation of the protein, which increase enzyme activity. (B) At medium protein concentration the MPO interaction with the surface is influenced by other proteins and is very active. (C) In high protein concentration media the MPO has less access to the surface and is less active either because of less MPO is bound to the surface or that the conformational change is less favorable in a crowded environment.

The particle coverage is important for enzyme activity. The activity of trypsin adsorbed to copper sulfide NPs is unaffected at low surface coverage whereas high surface coverage lowers the activity [39]. The opposite situation was shown for pyrophosphatase, as the activity was low at low enzyme coverage and high at high surface coverage [40]. The effect of surface coverage is attributed to the structural changes in the enzyme after binding to the surface [39,40]. The effect of NPs on MPO activity is likewise dependent on the total protein concentration as the presence of FCS or BSA affected the enzyme activity. This can be explained by that the interaction of MPO with the NPs is different if the surface area is partly covered by other proteins, which thereby limit or change how MPO binds to the surface, Fig 7. The 60 nm PS-COOH NPs, on their own, activate MPO. Adding BSA to the system at low concentrations increases the activation. At higher BSA concentrations, the activity is decreased. At low BSA concentrations, MPO may still bind to the particle surface but due to limited space on the surface, the binding mechanism is different compared to a situation where there is unlimited space to bind to. This leads to a more activated MPO. At higher BSA concentrations, MPO may be exchanged by BSA, and the inhibitory effect of BSA on MPO in solution is dominating.

Lowering the activity of the enzymes produced during the degranulation is one kind of effect that NPs in this study can have on the oxidative metabolism of neutrophils. In another perspective, the effect of NPs by enhancing the activity of the enzymes and overproduction of reactive oxygen species may lead to autoimmune pathology as observed in many inflammatory conditions [41]. NPs may affect the immune response in different ways [7]. For instance, activation of the complement system is a well-known effect of diverse NPs [42]. Our study shows that NPs can have a direct impact on the activity of immune effector enzymes. This emphasizes the need of further studies on the effects that NPs may have on enzymes mediating crucial biological pathways.

## Supporting information

S1 Appendix Includes text Table A, Figures A and B.
(DOCX)
