# Supplementary material for: Nanoparticle effect on neutrophil produced myeloperoxidase
Source: PLoS One. 2018 Jan 18;13(1):e0191445. doi: 10.1371/journal.pone.0191445 (PMC5773199; doi:10.1371/journal.pone.0191445)
Supplement: S1 Appendix — Includes text Table A, Figures A and B. (DOCX) [file pone.0191445.s001.docx]

**Nanoparticle effect on neutrophil produced myeloperoxidase**

E. Sanfins, A. Correia , S. Gunnarsson, M.Vilanova and T. Cedervall

**Z-potential of the nanoparticles**

**Table A.** Z potential of polystyrene and TiO_2_ nanoparticles.

| Nanoparticles | Z potential in PBS (mV) | | |
| --- | --- | --- | --- |
|  | 1 mg/ml | 0.1 mg/ml | 0.01 mg/ml |
| PS-COOH (26) | -39 ± 2 |  |  |
| PS-COOH (60) | -33 ± 3 |  |  |
| PS-COOH (220) | -38 ± 2 |  |  |
| PS-NH_2_ (56) | +26 ± 1 | +7 ± 2 |  |
| PS-NH_2_ (120) | -27 ± 1 | -9 -1 ± 1 | -5 -1 ± 1 |
| TiO_2_ (5) | -23 |  |  |

The zeta potential of the particles, diluted PBS at indicated particle concentrations, was measured using Malvern Zetasizer (Malvern Instruments Inc., England). All PS-COOH have a negative Z potential at 1 mg/ml particle concentration. The 56 nm PS-NH_2_ has a clear positive Z-potential whereas the Z-potential of 120 PS-NH_2_ is negative. However, for the amine modified particles we observed a sedimentation during the experiment, especially for the 120 PS-NH_2_, which likely affect the data. To decrease the sedimentation and optimize the measurements we diluted the amine modified particles 10 and 100 times. Dilution decreased the sedimentation but not totally. The Z-potential for both particle sizes changed into values that are considered to be neutral (± 20 mV).

**Interaction of MPO with different NPs**

The interactions between MPO and the NPs used in this study was confirmed by using a step wise sucrose gradient. After 1 h incubation, the MPO/NPs mixtures were loaded on top of a stepwise sucrose gradients in 1.5 ml eppendorfs tubes. The top sucrose is 10% in PBS and the bottom 40% in PBS and TMB, the MPO substrate. The samples were centrifuged at 18000 rpm for 30 min. Only MPO bound to the NPs will travel through the 10% sucrose solution and the protein NPs complexes stops at the 40% sucrose. The presence of MPO on the NPs is determined by the blue colour. All NPs bind to MPO (figure S1). There are apparent differences in the colour reaction between the different samples, but the assay is not qualitative as different NPs and MPO complexes may sediment differently into the 40% sucrose layer.


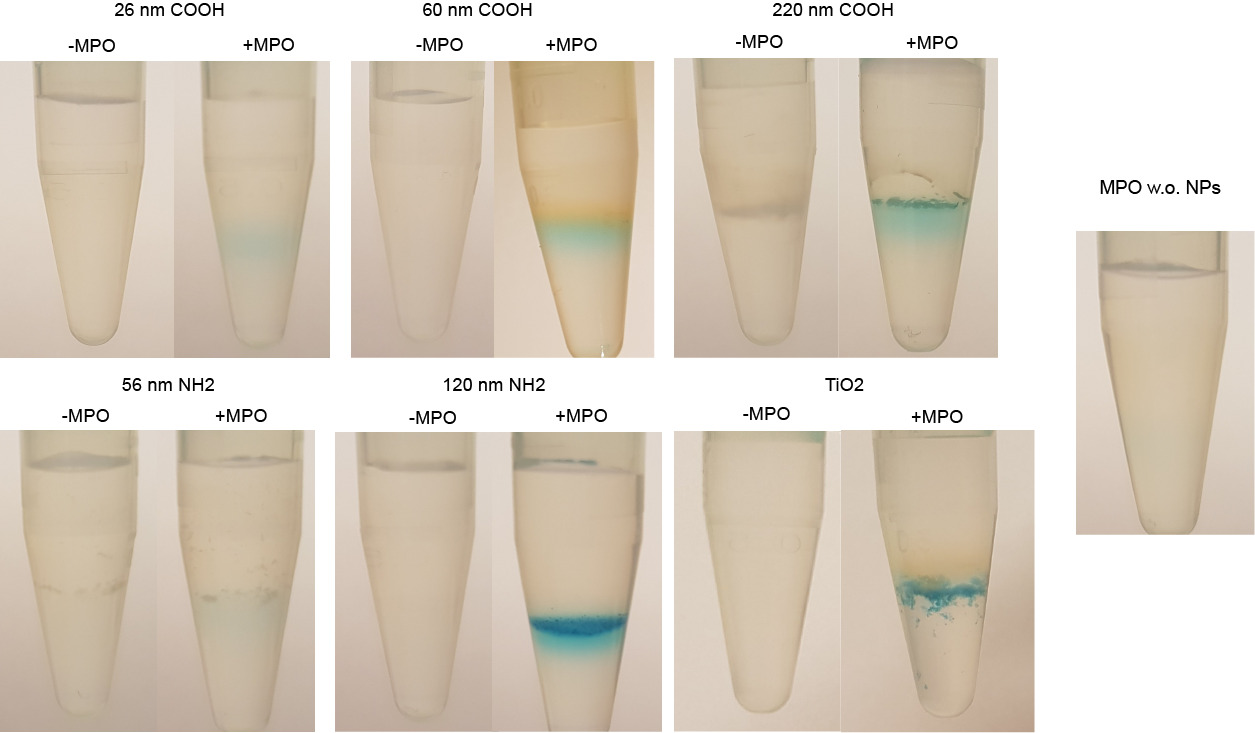


**Fig A.** **Interaction of all NPs with MPO control carried out with only MPO and without NPs**.

**Interaction between MPO and 60 nm PS-COOH in different concentrations of BSA**

To qualitatively determine the interaction between MPO and NPs in different concentrations of BSA the same assay with a step wise sucrose gradient as above was used. MPO, 1 µg, was mixed with BSA 0 to 9 mg/ml, before adding the nanoparticles, 1 mg/ml in PBS. As is seen in supplementary Figure 1, MPO is present on the NPs at all BSA concentrations. There seems to less MPO, due to weaker blue colour, at 3 and 9 mg/ml BSA. However, the assay is not quantitative, as for example, more BSA bound to the particles changes the complexes density and thereby the broadness of the band.


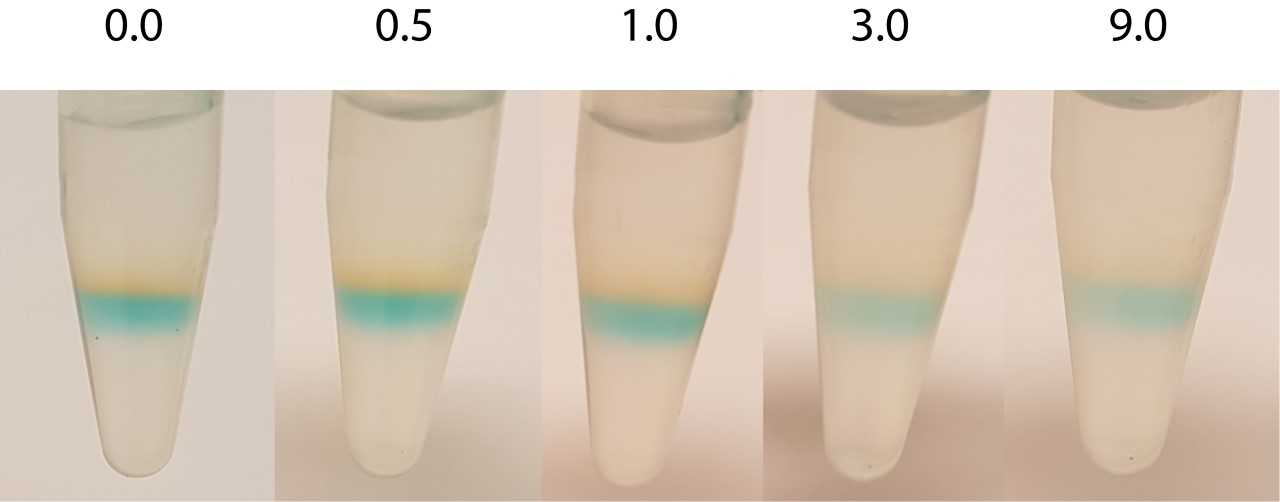


**Fig B.** **MPO interacts with 60 nm PS-COOH in different concentrations of BSA.** The BSA concentrations are from left to right 0, 0.5, 1, 3, and 9 mg / ml.
